# Heterogeneity of Genetic Damage in Cervical Nuclei and Lymphocytes in Women with Different Levels of Dysplasia and Cancer-Associated Risk Factors

**DOI:** 10.1155/2015/293408

**Published:** 2015-08-03

**Authors:** Carlos Alvarez-Moya, Mónica Reynoso-Silva, Alejandro A. Canales-Aguirre, José O. Chavez-Chavez, Hugo Castañeda-Vázquez, Alfredo I. Feria-Velasco

**Affiliations:** ^1^Cellular and Molecular Biology Department, Guadalajara University, Juarez 976, Colonia Centro, 44100 Guadalajara, JAL, Mexico; ^2^Unit of Medical and Pharmaceutical Biotechnology, CIATEJ, Avenida Normalistas 800, Colinas de la Normal, 44270 Guadalajara, JAL, Mexico; ^3^Dysplasias Unit, Institute of Cancerology Jaliscience, General Coronado 515, Colonia Centro, 44100 Guadalajara, JAL, Mexico; ^4^Mastitis and Molecular Diagnosis, Guadalajara University, Juarez 976, Colonia Centro, 44100 Guadalajara, JAL, Mexico

## Abstract

The comet assay can be used to assess genetic damage, but heterogeneity in the length of the tails is frequently observed. The aims of this study were to evaluate genetic damage and heterogeneity in the cervical nuclei and lymphocytes from patients with different levels of dysplasia and to determine the risk factors associated with the development of cervical cancer. The study included 97 females who presented with different levels of dysplasia. A comet assay was performed in peripheral blood lymphocytes and cervical epithelial cells. Significant genetic damage (*P* ≤ 0.05) was observed only in patients diagnosed with nuclei cervical from dysplasia III (NCDIII) and lymphocytes from dysplasia I (LDI). However, the standard deviations of the tail lengths in the cervical nuclei and lymphocytes from patients with dysplasia I were significantly different (*P* ≤ 0.0001) from the standard deviations of the tail lengths in the nuclei cervical and lymphocytes from patients with DII and DIII (NCDII, NCDIII and LDII, LDIII), indicating a high heterogeneity in tail length. Results suggest that genetic damage could be widely present but only manifested as increased tail length in certain cell populations. This heterogeneity could obscure the statistical significance of the genetic damage.

## 1. Introduction

Cervical uterine cancer (CCU) is the second most common cancer among women and is responsible for over 250,000 deaths annually [1]. CCU is related to human papillomavirus (HPV), which activates early genes E6 and E78 of the host giving rise to benign or premalignant cervical lesions. There are other etiological factors besides human papillomavirus (HPV) and its risk of occurrence varies from one population to another [2–6]. CCU begins as a mild hyperplasia (I) and then progresses through moderate hyperplasia (II), severe hyperplasia (III), and, ultimately, cervical carcinoma [6]. In women with cervical dysplasia, genomic instability has been observed and is manifested as chromosomal abnormalities, presence of micronuclei, sister chromatid exchange [7–10], and a significant increase in DNA damage in cervical epithelial nuclei [11]. Genetic instability is a necessary condition for cancer development and the accumulation of DNA damage is the molecular basis of cancer progression [12, 13] so genetic damage in dysplastic cells is an important criterion for the diagnosis of CCU [7]. Because both genetic damage and progression of dysplasia may be influenced by orden, quantity, and combination of exposure to various risk factors and, due to that several studies have reported higher frequencies of chromosomal damage in the lymphocytes of individuals exposed occupationally or environmentally [14, 15], it is totally possible to use as monitors of genetic damage to lymphocytes of patients with varying degrees of dysplasia and simultaneously visualize genetic damage in the nuclei of cells from dysplastic tissue.

The comet assay is frequently used to detect DNA damage because the method is sufficiently simple and sensitive to detect damage in individual cells. In this technique, cells embedded in agarose are placed on a microscope slide, detergents and high salts to lyse the cells and make DNA electrophoresis under alkaline or neutral conditions. Cells with an increased frequency of DNA double-strand breaks show increased migration of DNA toward the anode. The migrating DNA is quantitated by staining with ethidium bromide and by measuring the intensity of fluorescence at two fixed positions within the migration pattern using a microscope photometer [16, 17]. The comet tail is the result of the migration of DNA fragments, broken strands, and sites sensitive to alkali. Variations in the tail length of comet nuclei among cells in the same study indicate differences in the degree of damage [18]; however, although comet assay detects genetic damage which is an important criterion for diagnosing cancer risk, the frequent heterogeneity of tail lengths observed using the comet assay [18] could prevent the significant detection of genetic damage. In this work, genetic damage and heterogeneity in cervical nuclei and lymphocytes from patients with different levels of dysplasia were determined. Additionally, the risk factors associated with the development of cervical cancer were evaluated.

## 2. Materials and Methods

### 2.1. Reagents

All reagents were purchased from Sigma Chemical Co. (Guadalajara, Jalisco, México), except dimethyl sulphoxide (DMSO) and disodium EDTA, which were obtained from J.T. Baker (D.F., México).

### 2.2. Study Population

The study included a total of 97 women (sample size sufficient) diagnosed with different stages of cervical neoplasia who received a gynaecological check-up at the Hospital General de Occidente Zoquipan de la Secretaría de Salubridad del Estado de Jalisco in the period between 1 July 2012 and 30 June 2013. Subjects were coded and at the time of sampling to perform the study blinded. Details including the patients' age, whether they smoked or lived with a smoker, family history of cancer, medical treatment and exposure to environmental, and occupational or household chemicals were noted in a questionnaire at the time of sampling.

### 2.3. Sampling

Two types of samples were collected from each individual, as performed in Udumudi et al. [19]. Informed consent was obtained from the patients and the ethical guidelines of the hospital were followed. The two types of cell samples and their collection methods were as follows: (A) peripheral blood lymphocytes (PBL). For each individual, blood from a finger prick was collected in 2 conical tubes containing 10 mL of phosphate buffer solution (PBS) (160 mM NaCl, 8 mM Na_2_HPO_4_, 4 mM NaH_2_PO_4_, and 50 mM EDTA; pH 7) and immediately transported to the laboratory and centrifuged at 3000 rpm for 5 min. The supernatant was removed, and the pellet was suspended in PBS and immediately stored at 4°C until use. This small amount of whole blood was sufficient to study DNA damage using the comet assay and viability using the trypan blue exclusion test. The mean percentage of viability for each group was >89%: (B) cervical epithelial cells. The cervix was gently wiped with a sterile swab, and the shaped end of a wooden spatula was used to scrape the cervix in a 360° fashion. Two scrapings were collected from each patient. A sample from the first scraping was spread on a glass slide and fixed immediately in ethyl alcohol; this sample was used in a Pap smear test to determine the pathological status of the cervix. A sample from the second scraping was suspended in PBS and was later used in the comet assay to study DNA damage and the trypan blue exclusion test to determine cell viability. The samples were brought to the lab under cold conditions and processed within 1 h of sampling to avoid artefact damage to the DNA.

### 2.4. Classification of Samples

The Pap smear test was used because it is an ideal method to detect cervical neoplasia. Based on the results of the Pap smears, the women were divided into four categories: negative control (cervical cells and lymphocytes of healthy women without dysplasia), dysplasia low, dysplasia moderate, dysplasia severe, and cervical carcinoma in accordance with the Koss and Durfee cytomorphological classification [20]. The samples were graded as follows: nuclei cervical from women with dysplasia I (NCDI), dysplasia II (NCDII), and dysplasia III (NCDIII) and negative control (NCNC) and lymphocytes from dysplasia I (LDI), dysplasia II (LDII), and dysplasia III (LDIII) and negative control (LNC).

### 2.5. Comet Assay

To perform the comet assay, the cell suspension of cervical epithelial cells and lymphocytes was placed on different slides and mixed with low melting-point agarose at 37°C, to a final concentration of 0.7%. The mixture (100 *μ*L) was pipetted onto slides with 0.5% normal-melting-point agarose, to retain the agarose cell suspension. The drop containing the cells was covered with a glass cover slip (24 mm × 24 mm) and left at 4°C for 5 min. The cover slips were gently removed and the slides were then ready for processing. The alkaline comet assay was performed using the basic rationale of Singh et al. [21], with modifications.

To induce nuclear lysis and facilitate DNA unfolding, the slides were immersed in a lysis buffer (2.5 M NaCl, 100 mM Na_2_EDTA, 10 mM Tris-HCl, 1% sodium lauryl sarcosine, 1% Triton X-100, and 10% DMSO, pH 10) for 2 h at 4°C. The slides were then placed in a horizontal electrophoresis system with a high pH buffer (30 mM NaOH, 1 mM Na_2_EDTA, pH 13) for 45 min to allow unwinding of the DNA prior to electrophoresis, which was performed for 15 min at 1.0 V/cm with an accompanying amperage of approximately 300 mA. The same electrophoresis unit and power supply were used throughout the study [22]. To avoid additional DNA damage, all of the steps described above were performed under yellow light. Following electrophoresis, the slides were gently washed to remove the alkali and then were immersed in a neutralisation buffer (0.4 M Tris-Base, pH 7.5) for 5 min. Gels were stained with ethidium bromide (100 mL at 20 mg/mL) for 3 min and then rinsed three times with distilled water. The preparation was then cover-slipped. The fluorescence microscopy used to examine the slides employed a light microscope equipped with a 515–560 nm excitation filter. Comets were observed at 40x magnification, and migration was determined by visually scoring the tail length according to published protocols [22]. Approximately 50 comets per slide and two slides for each individual, including controls, were evaluated.

### 2.6. Statistical Analysis

The slides were coded at the time of preparation, scoring, and analysis. The results were expressed as the mean ± SD and were analyzed by one-way analysis of variance (ANOVA) using the CoStat program [23]. All of the study groups were compared with the corresponding negative control using the Dunnett test. Pearson's correlation was performed with SPSS 10.0 (Software, SPSS Inc.). Fifty cells were evaluated from each individual slide. A value of *P* ≤ 0.05 indicated significance.

## 3. Results

**Figure 1:**
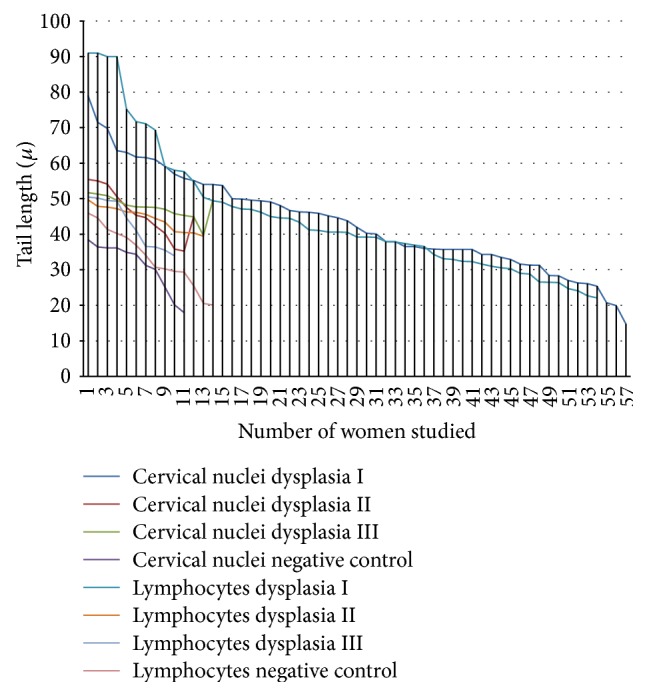
Distribution of the average tail length of cervical nuclei and lymphocytes from women with different degrees of dysplasia. On the *x*-axis the number women screened is displayed. The color refers to the cell type studied and the level of dysplasia. The *y*-axis indicates the average tail length of lymphocytes or cervical nucleus in each of women.

The distribution and average tail lengths of lymphocytes and cervical nuclei from women with different levels of dysplasia are presented in Figure 1. All of the study groups showed heterogeneity in the average migration tail length, although cervical nuclei and lymphocytes from the DI group showed a higher degree of heterogeneity. The correlation coefficients between the average migration of the cervical nuclei and lymphocytes from women with varying degrees of dysplasia are shown in Table 1. NCDI and LDI showed the highest degree of correlation (0.9606). NCDII-LDII, NCDIII-LDIII, and NCNC-LNC had correlations of 0.8735, 0.9360, and 0.8856, respectively.

**Figure 2:**
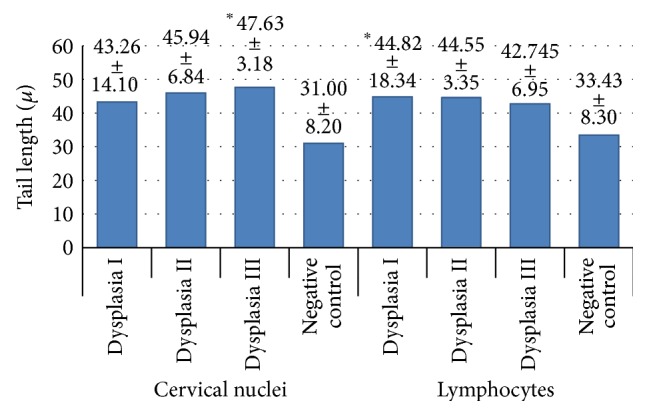
Average tail length (genetic damage) of cervical nuclei and lymphocytes from women with different grades of dysplasia. The number in the bars corresponds to the average tail length of cervical cells or nuclei from women with a certain level of dysplasia. Cervical cells and nuclei were placed separately. The negative control is also visualized. ^*^ *P* ≤ 0.05.  ^*^Bartlett's test suggests that the SD in both cervical tissue nuclei and lymphocytes from cervical dysplasia I are significantly different (*P* ≤ 0.0001) from those of lymphocytes and cervical tissue nuclei from dysplasia II and III.

Averages of the DNA migration for both the lymphocytes and the cervical nuclei were obtained and are presented in Figure 2 and the comparisons with the corresponding negative controls were statistically significant (*P* ≤ 0.05) only for NCDIII and LDI (Figure 2). However, the standard deviations of the cervical nuclei and lymphocytes from the dysplasia I group were significantly different (*P* ≤ 0.0001) from the cervical nuclei and lymphocytes from the DII and DIII groups. The comparison between negative controls showed no significant difference.

Table 2 shows the percentages of the participants who were exposed to various risk factors for cervical cancer (except for the presence of HPV). Women reported having exposure to multiple risk factors; Table 2 also shows the sum of the percentages of women exposed to each risk factor.

## 4. Discussion

The HPV is considered to be the most important risk factor in the aetiology of cervical cancer [24, 25]; however DNA and cells in the human body are constantly exposed to oxidative attack by both exogenous and endogenous agents that are capable of inducing genetic damage associated with cancer development [26] and may play an important role in cervical carcinogenesis; these agents should thus be considered as risk factors: smoking and other factors also increase the risk of this disease through oncogenic activation [27–29]. Additionally, it has been reported that there is a synergistic effect between smoking and HPV infection on cervical carcinogenesis [30, 31]. Considering that in our study the percentage of women who smoked was low (15.8%) and that all the patients showed some form of dysplasia, it follows that there are other risk factors that collaborate with HPV, as also reported by Schiffman and Castle [27]. The data in Table 2 show a high percentage of nonsmoking women who were exposed to several other risk factors. The order of the rates of exposure (home exposure to chemicals > living with a smoker > takes medications > environmental exposure to chemicals > family history of cancer > smoker > occupational exposure to chemicals > recent exposure to X-ray) suggested that some risk factors and their combinations could induce cervical carcinogenesis, although we were unable to determine the extent of genetic damage caused by each factor.

Strikingly, in addition to the 15.8% of participants who were smokers, 40.9% of the women studied lived with smokers; therefore, the percentage of the participants who were exposed to cigarette smoke was actually much higher (Table 2). A similar situation could be observed with the other risk factors; then it is possible to observe an increase in both heterogeneity and genetic damage in women exposed to different risk factors; further the frequency and duration of the exposure to various chemical agents were varied. Additionally, several other indirect factors, including low socioeconomic status, early marriage and first child, multiparity, poor personal hygiene, and genital infections, which can also increase the exposure to risk factors and consequently lead to damage genetic, were not considered in this study [31]. All of the above risk factors induce different amounts of genetic damage in the cervical nuclei and lymphocytes because of the differential exposure of these cell types, which contributes to the substantial heterogeneity of tail length, for example, a large difference in tail length since 15–80 microns in NCDI were observed. Similar situation occurred in LDI with variations since 22–90. As shown, the group of patients with dysplasia I was the most frequent and large during the time period of this studio. The cases of patients with dysplasia II and dysplasia III were less frequent, but also great heterogeneity of length tail was observed in both lymphocytes and cervical nuclei. Importantly, the similarity of migration and heterogeneity between lymphocytes and cervical nuclei (see correlation coefficients in Table 1) highlighting the case of LDI and NCDI (0.9606), LDII-CNDII (0.8735), LDIII-CNDIII (0.9360), and negative controls for lymphocytes and cervical nuclei (0.8856). Therefore it is understood as the usefulness of lymphocytes as biomonitors of genetic damage in cervical dysplasia (Figure 1). Another factor that contributes to the heterogeneity of tail lengths is the difference in DNA damage repair between the cell types studied, as previously reported [32]. There are also established differences in diet associated with the development of different types of cancer [33]. Ostling and Johanson demonstrated* in vitro* that these differences can contribute to the aetiology of heterogeneity in the same population of treated cells [34]. Those authors hypothesized that the heterogeneity of the tail length was the result of differential induction of genetic damage among cells. Differential genetic damage and genetic heterogeneity were subsequently demonstrated in animal cells from tumor biopsies and clinical models, explaining the resistance of some tumors to cancer treatment [35–37].

Because of the heterogeneity of the tail length, many authors have suggested that different types of comets should be recognized and considered separately [38]. In our study, several types of comets were observed, which contributed to the heterogeneity; it was clear that there were different cells or nuclei with different magnitudes of genetic damage* in vivo* [16]. In this context, the age of the lymphocytes and nuclei studied is an important factor to be considered [39–42]; increased age is correlated with the heterogeneity in the average migration (see negative control (Figure 1)). The ability of cells to repair DNA is lower in older lymphocytes compared with younger cells [39, 43].

Udumudi et al. [19] studied the relationship between genetic damage and the development of cervical neoplasia in nuclei and lymphocytes from women with cervical cancer using the comet assay and showed that genetic damage was proportional to the degree of dysplasia [13]. In our study, only samples from the NCDIII and LDI groups had sufficient cumulative damage to be detected by the comet assay (Figure 2). The other groups did not show a significant difference with the corresponding negative controls; however, cervical nuclei appeared to have damage proportional with the degree of dysplasia (Figure 2). The LDI group showed significant genetic damage (*P* ≤ 0.05). In the LDII and LDIII groups, despite an increase in migration that approached significance, there was no statistically significant difference in genetic damage (LDI 44.82 *μ*m, LDII 44.55 *μ*m, and LDII 42.74 *μ*m). Genetic damage could be present but only manifested as increased tail length in certain cell populations, as has been found in previous studies [34]. This possibility could have led to the dilution of the statistical significance of the migration averages (Figure 2) in our study, so our data are different from those reported by Udumudi et al. [19].

The comet assay is very efficient and allows for the direct visualization of genetic damage in individual cells [21], typically by measuring the average migration of the tail (average genetic damage). In our study, the average genetic damage is not significant in all of the study groups (Figure 2). Some populations of cells showed more damage, which caused heterogeneity in the migration of the tail length among study subjects (Figure 1). Bartlett's test suggests that the SD obtained from the CNDI group is significantly different (*P* ≤ 0.0001) from the SD of the nuclei from the cervical dysplasia II and III groups, and this difference manifests itself in the high heterogeneity in tail length. Heterogeneity could obscure the statistical significance of the damage. The NCDI group appears to have more genetic damage than the NCDII and NCDIII groups (Figure 1), but the average genetic damage is greater in the NCDIII group (Figure 2). The similar levels of genetic damage and high correlation coefficients between lymphocytes and cervical nuclei suggest that lymphocytes may serve as sentinel cells (Table 1). However, the presence of substantial heterogeneity in the tail length should be considered. Ideally, only the cells in each sample that have increased genetic damage would be considered in the measurement, including those in the negative controls.

## 5. Conclusion

There is differential genetic damage manifested as increased heterogeneity in the lengths of the tail length of cervical nuclei and peripheral blood lymphocytes in women with cervical dysplasia. This observed heterogeneity may confound the detection of significant genetic damage.

The comet assay is an excellent tool for determining the presence of genetic damage. However, to contribute to the reduction of heterogeneity in tail length, only cells with an increased tail length would ideally be included in the measurements.

## Figures and Tables

**Table 1:** Correlation coefficients between the average tail length of cervical nuclei and lymphocytes from women with different levels of dysplasia. The bold numbers indicate correlation coefficients between lymphocytes and cervical cells in the same grade of dysplasia.

	NCDI	NCDII	NCDIII	NCNC
LD I	**0.9606**	0.9039	0.7473	0.8996
LD II	0.9080	**0.8735**	0.9090	0.9793
LD III	0.8284	0.9579	**0.9360**	0.8751
LNC	0.9115	0.8632	0.7311	**0.8856**

**Table 2:** Cumulative percentage of women exposed to one (first-line) or more (second-line) onward risk factors for cervical cancer. Percentages of women exposed to more risk factors were obtained by adding individual percentages of exposure.

	Smoker (S)	Living with a smoker (LS)	Takes medications (TM)	Recent exposure to X-ray (RERX)	Family history of cancer (FHC)	Occupational exposure to chemicals (OECH)	Home exposure to chemicals (HECH)	Environmental exposure to chemicals (EECH)
	**15.8** ^*^	**40.9** ^*^	**29.5** ^*^	**2.2** ^*^	**20.4** ^*^	**6.8** ^*^	**70.4** ^*^	**22.7** ^*^
Smoker		56.7^**^	45.3^**^	18^**^	36.2^**^	22.6^**^	86^**^	38.5^**^
Living with a smoker			70.4^**^	43.1^**^	61.3^**^	47.7^**^	111.3^**^	63.6^**^
Takes medications				31.7^**^	49.9^**^	30.3^**^	99.9^**^	52.2^**^
Recent exposure to X-ray					22.6^**^	9^**^	72.6^**^	24.9^**^
Family history cancer						27.2^**^	90.8^**^	43.1^**^
Occupational exposure to chemicals							77.2^**^	29.5^**^
Home exposure to chemicals								93.1^**^

Note: percentages correspond to the responses of the women studied regarding the types of exposure to cancer risk factors. Clearly, there is simultaneous exposure to more than one risk factor.

^*^Refer to exposure to an agent.

^**^Refer to the simultaneous exposure to two or more agents.
